# Investigation of a Fiberoptic Device Based on a Long Period Grating in a Ring Resonator

**DOI:** 10.3390/s16091357

**Published:** 2016-08-24

**Authors:** Cinzia Corcione, Benedetto Troia, Francesco De Leonardis, Vittorio M. N. Passaro

**Affiliations:** Photonics Research Group, Department of Electrical and Information Engineering, Politecnico di Bari, via E. Orabona n. 4, Bari 70125, Italy; cinziac.corcione@gmail.com (C.C.); benedetto.troia@poliba.it (B.T.); francesco.deleonardis@poliba.it (F.D.L.)

**Keywords:** long period grating (LPG), ring resonator (RR), transfer matrix method (TMM), optical fiber sensor, strain, refractive index (RI)

## Abstract

A fiberoptic architecture based on a ring resonator (RR) including a typical long period grating (LPG) was investigated. The interactions between the fundamental core mode (*LP*_01_ or *HE*_11_) coupled to the RR and the cladding mode (*LP*_08_), excited into the cavity by means of the LPG, allow a peculiar spectral response characterized by two splitting resonances to be achieved. The new LPGRR architecture is investigated theoretically and a mathematical modelling based on the transfer matrix method (TMM) is proposed. The theoretical results are compared with the experiments measured by an open-loop LPG, while the performance of the relative LPGRR was estimated by a theoretical parametric analysis. Finally, an overview of the possible LPGRR sensing applications is provided by investigating the features of a strain sensor operating in different environmental conditions.

## 1. Introduction

Long period fiber gratings (LPGs) have attracted much attention in recent years and become the subject of intense research for sensing and communication due to peculiar advantages such as immunity to electromagnetic interference, high sensing resolution, low insertion loss, low back-reflections, compactness, and large-scale and low-cost fabrication, among many others [1,2,3,4,5,6,7]. Currently, LPGs are important optical fiber passive components, and the accuracy of the numerical tools used for the calculation of wavelength-dependent transmittance has become a key factor, as the LPG operating sensing principle is closely related to a wavelength readout scheme [8,9].

In this paper we propose, for the first time to the best of our knowledge, the mathematical model of a device named as long period grating ring resonator (LPGRR), where the LPG is assumed to be inscribed in a fiber ring resonator (RR). In fact, LPGs have been always studied and discussed as stand-alone devices, in open-loop configurations, to operate as strain, temperature, and refractive index sensors with maximum sensitivities equal to 30.31 nm/1000 µε, 0.861 nm/°C, [10], and 10^3^–10^4^ nm/RIU [11,12], respectively or as band-rejection filters [13]. The only exception consists in a microwave photonic filter based on a fiber cladding-mode coupler, where the cladding-mode generated by an external LPG is injected into a RR and coupled out from the device where another identical LPG is placed outside the RR and used to convert the cladding mode into a core mode [14].

The transfer matrix method (TMM) has been implemented to model and simulate the LPGRR assumed to be based on a three-layer step-index fiber geometry, in agreement with Erdogan’s theory used in this paper to calculate the cladding and core optical field components and core-to-cladding coupling coefficients in the LPG fiber [15]. The mathematical model is demonstrated to be flexible, fast and accurate, and allows the coupled-mode theory (CMT) to be implemented for the design of directional couplers characterized by fiberoptic input/output bus and the LPGRR.

The effective refractive indexes of cladding and core fiberoptic modes have been calculated by using the finite element method (FEM) [16], instead of the graphical method based on Bessel functions widely used in the literature [15].

The paper is organized as follows. In Section 2, the TMM-based modeling of the LPGRR is presented. In Section 3, the simulation results are shown and discussed describing the LPGRR operation and unique spectral peculiarities. Further, LPG geometrical and optical features such as the length and the core-to-cladding coupling coefficients have been varied to perform parametric simulations. Theoretical results have been compared to an open-loop LPG experimental spectrum, demonstrating a very good agreement. Section 4 investigates the LPGRR-based strain sensing performances as a function of different environmental conditions simulated as cover refractive index variations. Section 5 summarizes the conclusions.

## 2. Modeling

The fiberoptic resonant architecture consists of a fiber racetrack cavity in which a conventional LPGRR is included. In particular, LPGRR can be simply modeled by the TMM, scattering matrix and CMT. Further, a linearly polarized field that corresponds to the core mode is coupled to the RR by means of a fiber directional coupler and to the cladding mode by the LPG, provided that the phase matching condition (PMC) is satisfied [13,15].

**Figure 1:**
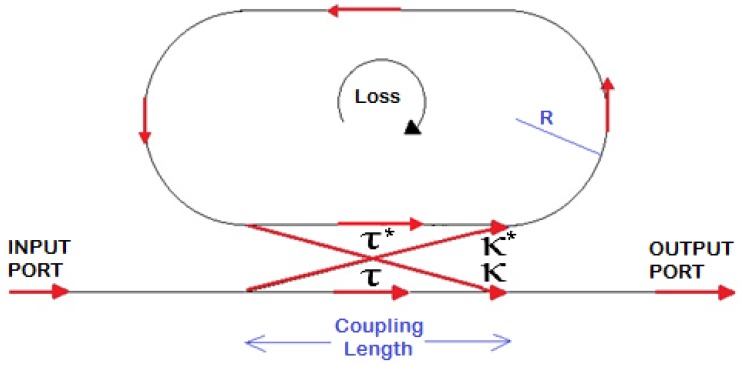
Sketch of a racetrack resonator characterized by a directional coupler (the * denotes the conjugated complex value of *τ* and *κ*, respectively).

The fiber RR is modeled in Figure 1, where the arrows indicate the optical signal propagation path, *R* is the ring radius, and *τ* and *κ* are the directional coupler transmission and coupling factors, respectively. In particular, several assumptions can be made to investigate the device operation and performance. For example, the excitation of a propagating unidirectional resonant single mode within only one polarization state, as well as lossless coupling, is assumed. Further, it is supposed that none of the waveguide segments and coupler elements can couple waves of different polarization states, and that all the loss contributions occurring along the light propagation in the ring resonator filter are included in the attenuation constant. In particular, the field profiles and the effective refractive indexes of the supermodes associated with the fundamental and selected cladding modes allows the analytical calculation of the complex transmission and coupling factors *τ* and *κ* to be performed by evaluating the overlap integrals at their respective ports [17,18,19,20].

**Figure 2:**
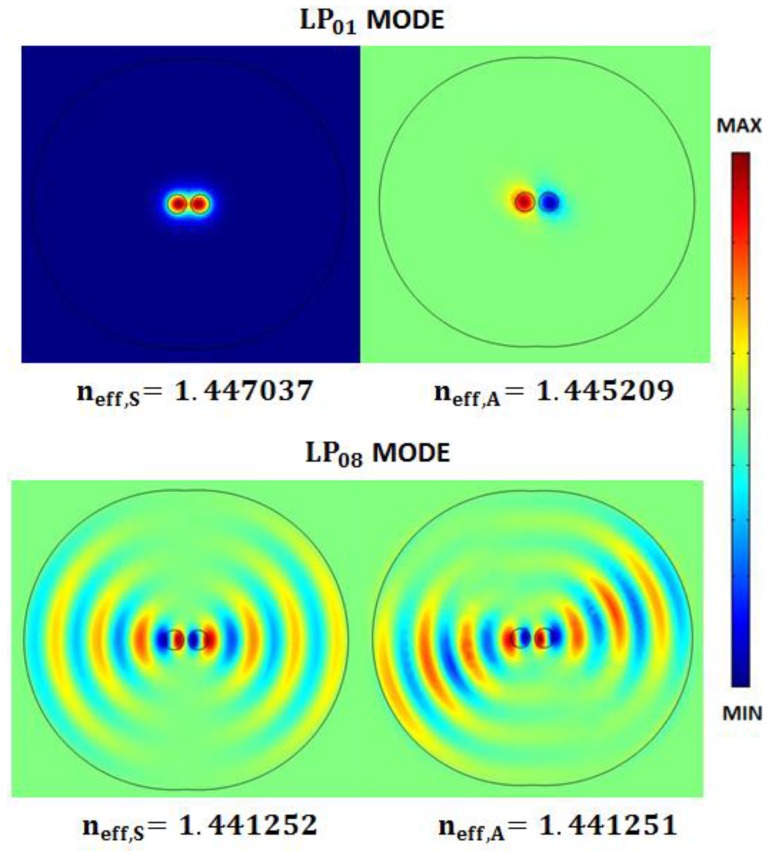
Symmetric and antisymmetric supermodes profiles and effective refractive indexes for *LP*_01_ core mode and *LP*_08_ cladding mode, respectively, simulated by FEM at 1550 nm considering two coupled fibers with core-to-core gap, *DC_gap_*, equal to 10 µm.

Figure 2 shows the electric components of the TE symmetric and antisymmetric supermodes of the directional coupler calculated by a full-vectorial finite element method and compares the supermode profiles obtained directly from the mode solver for *LP*_01_ core and *LP*_08_ cladding modes. Further details on fiberoptics geometrical and optical parameters are reported in Table 1 (Section 3).

**Figure 3:**
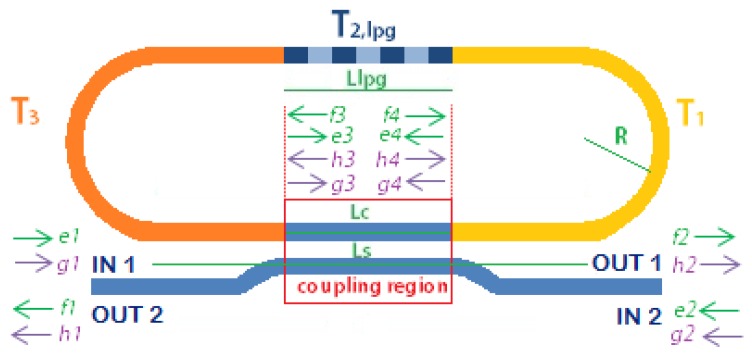
Generic 4-port scheme of LPGRR subdivided in elementary sections to derive the total transfer matrix as the product of individual matrices with core (green) and cladding (violet) fields directed according to the arrows’ directions.

The total transfer matrix is given by the product of the matrices corresponding to each section in which the device has been divided, in the reverse numbered order as indicated by the subscripts in Equation (1) and shown in Figure 3:

(1) $$T_{LPGRR}=T_{3}\cdot T_{2,lpg}\cdot T_{1}=\left[\begin{array}{c} T_{11}\\ T_{21} \end{array}\;\begin{array}{c} T_{12}\\ T_{22} \end{array}\right]$$

In Equation (1), *T*_2,*lpg*_ is the LPG scattering matrix and *T*_1_ = *T*_3_ are the scattering matrices associated with the highlighted sections of the cavity (see Figure 3). Figure 3 also shows the four-port representation of the LPGRR with inputs and outputs field components used to develop the analysis, i.e., *e_i_* and *f_i_* the core components, *g_i_* and *h_i_* the cladding components at the *i*-th port (*i* = 1, 2, 3, 4) with the directions of propagation indicated by the arrows.

The individual scattering matrices are defined as in Equations (2) and (3):

(2) $$T_{lpg}=\left[\begin{array}{c} T_{LPG}\\ O \end{array}\;\begin{array}{c} O\\ {T_{LPG}}^{-1} \end{array}\right]$$

(3) $$T_{1}=T_{3}=\left[\begin{array}{c} T_{fiber}\\ O \end{array}\;\begin{array}{c} O\\ {T_{fiber}}^{-1} \end{array}\right]$$

where *O* is a 2 × 2 zero matrix, *T_LPG_* is a 2 × 2 matrix defined as in Equation (4):

(4) $$T_{LPG}=\left[\begin{array}{c} e^{-j\left(\beta_{co}+\beta_{cl}+\frac{\pi }{\Lambda }\right)L_{lpg}}\\ 0 \end{array}\;\begin{array}{c} 0\\ e^{-j\beta_{co}\left(+\beta_{cl}-\frac{\pi }{\Lambda }\right)L_{lpg}} \end{array}\right]\cdot \left[\begin{array}{c} \cos\left(\gamma L_{lpg}\right)-\frac{j\delta }{\gamma }\sin\left(\gamma L_{lpg}\right)\\ -e^{j\phi }\frac{k_{co-cl}}{\gamma }\sin\left(\gamma L_{lpg}\right) \end{array}\;\begin{array}{c} e^{j\phi }\frac{k_{co-cl}}{\gamma }\sin\left(\gamma L_{lpg}\right)\\ \cos\left(\gamma L_{lpg}\right)+\frac{j\delta }{\gamma }\sin\left(\gamma L_{lpg}\right) \end{array}\right]$$

and

(5) $$T_{fiber}=\left[\begin{array}{c} \alpha \;e^{-j\beta_{co}L}\\ 0 \end{array}\;\begin{array}{c} 0\\ \alpha \;e^{-j\beta_{cl}L} \end{array}\right]$$

In Equations (4) and (5), ϕ is the grating phase, $\gamma =\sqrt{\delta^{2}+{k_{co-cl}}^{2}}$, with *δ* the detuning parameter, *L_lpg_* the LPG length, *k_co-cl_* is the core-to-cladding mode coupling coefficient, *β_co_* and *β_cl_* are the propagation constants of core and cladding modes, respectively [15]. Moreover, Λ is the grating period, *α* represents the cavity losses and *L* is the length of the orange and yellow colored bend and straight symmetric sections calculated as in Equation (6):

(6) $$L=\frac{L_{s}-L_{c}}{2}+\pi R+\frac{L_{s}-L_{lpg}}{2}$$

In Equation (6), *L_s_* is the physical length of the coupler, *L_c_* is the coupling length, and *R* is the racetrack radius as already defined in Figure 3.

In the coupling region, output fields (i.e., *f_i_* and *h_i_*) are related to the input fields (i.e., *e_i_* and *g_i_*) by the transmission and coupling coefficients as represented by the Equations (7)–(10), where τ, τ_c_ and κ, κ_c_ are the transmission and coupling coefficients for core and cladding modes, respectively.

(7) $$\left[\begin{array}{c} f_{1}\\ h_{1} \end{array}\right]=\left[\begin{array}{c} \tau e^{j\beta_{co}L_{c}}\\ 0 \end{array}\;\begin{array}{c} 0\\ \tau_{c}e^{j\beta_{cl}L_{c}} \end{array}\right]\cdot \left[\begin{array}{c} e_{2}\\ g_{2} \end{array}\right]+\left[\begin{array}{c} -j\kappa e^{j\beta_{co}L_{c}}\\ 0 \end{array}\;\begin{array}{c} 0\\ -j\kappa_{c}e^{j\beta_{cl}L_{c}} \end{array}\right]\cdot \left[\begin{array}{c} e_{4}\\ g_{4} \end{array}\right]$$

(8) $$\left[\begin{array}{c} f_{2}\\ h_{2} \end{array}\right]=\left[\begin{array}{c} \tau e^{j\beta_{co}L_{c}}\\ 0 \end{array}\;\begin{array}{c} 0\\ \tau_{c}e^{j\beta_{cl}L_{c}} \end{array}\right]\cdot \left[\begin{array}{c} e_{1}\\ g_{1} \end{array}\right]+\left[\begin{array}{c} -j\kappa e^{j\beta_{co}L_{c}}\\ 0 \end{array}\;\begin{array}{c} 0\\ -j\kappa_{c}e^{j\beta_{cl}L_{c}} \end{array}\right]\cdot \left[\begin{array}{c} e_{3}\\ g_{3} \end{array}\right]$$

(9) $$\left[\begin{array}{c} f_{3}\\ h_{3} \end{array}\right]=\left[\begin{array}{c} \tau e^{j\beta_{co}L_{c}}\\ 0 \end{array}\;\begin{array}{c} 0\\ \tau_{c}e^{j\beta_{cl}L_{c}} \end{array}\right]\cdot \left[\begin{array}{c} e_{4}\\ g_{4} \end{array}\right]+\left[\begin{array}{c} -j\kappa e^{j\beta_{co}L_{c}}\\ 0 \end{array}\;\begin{array}{c} 0\\ -j\kappa_{c}e^{j\beta_{cl}L_{c}} \end{array}\right]\cdot \left[\begin{array}{c} e_{2}\\ g_{2} \end{array}\right]$$

(10) $$\left[\begin{array}{c} f_{4}\\ h_{4} \end{array}\right]=\left[\begin{array}{c} \tau e^{j\beta_{co}L_{c}}\\ 0 \end{array}\;\begin{array}{c} 0\\ \tau_{c}e^{j\beta_{cl}L_{c}} \end{array}\right]\cdot \left[\begin{array}{c} e_{3}\\ g_{3} \end{array}\right]+\left[\begin{array}{c} -j\kappa e^{j\beta_{co}L_{c}}\\ 0 \end{array}\;\begin{array}{c} 0\\ -j\kappa_{c}e^{j\beta_{cl}L_{c}} \end{array}\right]\cdot \left[\begin{array}{c} e_{1}\\ g_{1} \end{array}\right]$$

In particular, by defining $A=\left[\begin{array}{c} \tau e^{j\beta_{co}L_{c}}\\ 0 \end{array}\;\begin{array}{c} 0\\ \tau_{c}e^{j\beta_{cl}L_{c}} \end{array}\right]$, and $B=\left[\begin{array}{c} -j\kappa e^{j\beta_{co}L_{c}}\\ 0 \end{array}\;\begin{array}{c} 0\\ -j\kappa_{c}e^{j\beta_{cl}L_{c}} \end{array}\right]$, and considering the definition of the LPGRR transfer matrix (i.e., Equation (1)), the incoming and outgoing fields at the 3rd port can be calculated as in Equations (11) and (12), respectively:

(11) $$\left[\begin{array}{c} e_{3}\\ g_{3} \end{array}\right]=T_{11}\left[\begin{array}{c} f_{4}\\ h_{4} \end{array}\right]+T_{12}\left[\begin{array}{c} e_{4}\\ g_{4} \end{array}\right]$$

(12) $$\left[\begin{array}{c} f_{3}\\ h_{3} \end{array}\right]=T_{21}\left[\begin{array}{c} f_{4}\\ h_{4} \end{array}\right]+T_{22}\left[\begin{array}{c} e_{4}\\ g_{4} \end{array}\right]$$

Then, by substituting Equation (10) in Equations (11) and (12), Equations (13) and (14) can be derived:

(13) $$\left[\begin{array}{c} e_{3}\\ g_{3} \end{array}\right]=T_{11}\left\{\;B\cdot \left[\begin{array}{c} e_{1}\\ g_{1} \end{array}\right]+A\cdot \left[\begin{array}{c} e_{3}\\ g_{3} \end{array}\right]\right\}+T_{12}\left[\begin{array}{c} e_{4}\\ g_{4} \end{array}\right]$$

(14) $$\left[\begin{array}{c} f_{3}\\ h_{3} \end{array}\right]=T_{21}\left\{\;B\cdot \left[\begin{array}{c} e_{1}\\ g_{1} \end{array}\right]+A\cdot \left[\begin{array}{c} e_{3}\\ g_{3} \end{array}\right]\right\}+T_{22}\left[\begin{array}{c} e_{4}\\ g_{4} \end{array}\right]$$

Finally, Equation (15) is also reported as calculated by Equation (14):

(15) $$\left[\begin{array}{c} e_{4}\\ g_{4} \end{array}\right]=N_{1}B\left[\begin{array}{c} e_{2}\\ g_{2} \end{array}\right]-N_{2}\left[\begin{array}{c} e_{1}\\ g_{1} \end{array}\right]-N_{3}\left[\begin{array}{c} e_{3}\\ g_{3} \end{array}\right]$$

where $N_{1}={\left(T_{22}-A\right)}^{-1}$, $N_{2}=N_{1}T_{21}B$, $N_{3}=N_{1}T_{21}A$, resulting in $\left[\begin{array}{c} e_{3}\\ g_{3} \end{array}\right]=L_{2}\left[\begin{array}{c} e_{1}\\ g_{1} \end{array}\right]+L_{3}\left[\begin{array}{c} e_{2}\\ g_{2} \end{array}\right]$ and $\left[\begin{array}{c} e_{4}\\ g_{4} \end{array}\right]=(N_{1}B-N_{3}L_{3})\;\left[\begin{array}{c} e_{2}\\ g_{2} \end{array}\right]-(N_{2}+N_{3}L_{2})\left[\begin{array}{c} e_{1}\\ g_{1} \end{array}\right]$, where $L_{2}=L_{1}^{-1}\left(T_{11}B-T_{12}N_{2}\right)$, and $L_{3}=L_{1}^{-1}T_{12}N_{1}B$, $L_{1}=\left(I-T_{11}A+T_{12}N_{3}\right)$.

By imposing the initial conditions *e*_1_ = 1, *e*_2_ = *g*_1_ = *g*_2_ = 0 (note that because of these assumptions *T_lpg_*, *T_i_*, *T_fiber_* and the matrices in Equations (7)–(10) are all diagonal), which means that there is only one input port and the input field is only that of the core mode (the cladding mode is generated by the LPG), the reflected components are given by Equation (16):

(16) $$\left[\begin{array}{c} f_{1}\\ h_{1} \end{array}\right]=(A+BN_{1}B-BN_{3}L_{3})\left[\begin{array}{c} e_{2}\\ g_{2} \end{array}\right]-B(N_{2}+N_{3}L_{2})\left[\begin{array}{c} e_{1}\\ g_{1} \end{array}\right]$$

The transmitted components can be calculated as in Equation (17):

(17) $$\left[\begin{array}{c} f_{2}\\ h_{2} \end{array}\right]=\left(A+BL_{2}\right)\left[\begin{array}{c} e_{1}\\ g_{1} \end{array}\right]+BL_{3}\left[\begin{array}{c} e_{2}\\ g_{2} \end{array}\right]$$

## 3. Results

**Figure 4:**
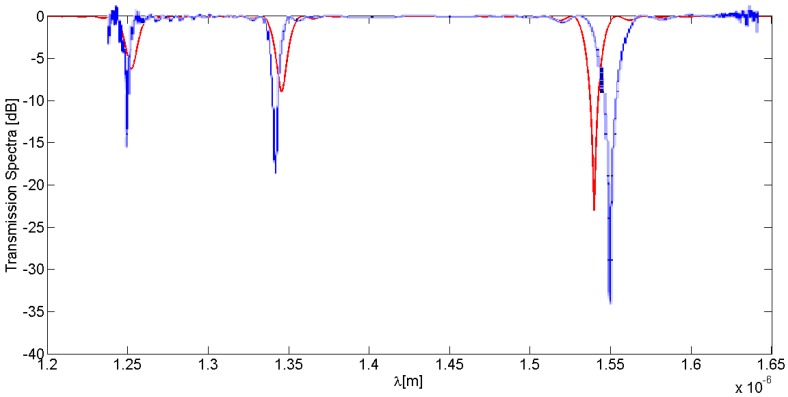
Comparison between theoretical (red curve) and experimental (blue curve) LPG spectral response.

A 3 cm-long LPG with a period of 311 µm and an average index change of the grating area equal to σ = 4 × 10^−4^ is assumed to be written in a quarter of a single-mode optical fiber ring resonator. This period satisfies the PMC [15] for LP_08_ cladding mode at the resonance wavelength of 1539.8 nm. The SMF-28 fiber and LPGRR design parameters used in the simulations are summarized in Table 1, while the transmission and coupling coefficients for both modes are obtained by using the Supermodes Theory in a 6.55 cm-long coupling region and listed in Table 2. The LPG has been written in the SMF-28 fiber by classical UV laser exposure through an amplitude mask. Experimental results have been achieved by measuring the transmission spectrum by an OSA, are plotted in Figure 4 and compared with theory.

A good agreement between theoretical and experimental spectral distributions of the open-loop LPG has been achieved as demonstrated in Figure 4, where a fitting core-to-cladding coefficient equal to 50 m^−1^ has been used in accordance with the LPG fabrication. Actually, a disalignment between experimental and theoretical LPG spectral resonances can be observed. However, some reasons for this behaviour can be identified. First, the theoretical calculations are based on the weakly guiding approximation that predicts the dispersion of the core and cladding modes with a limited accuracy. Second, the average value of the UV-induced grating was not rigorously constant, as assumed in the calculations, due to slightly different UV exposure times. Nevertheless, the experimental agreement with the predicted trends is quite good, with only very small discrepancies, quantifiable in a Δλ respectively equal to ~6 nm for the short wavelengths (e.g., 1250 and 1350 nm) and ~10.2 nm at 1550 nm.

**Figure 5:**
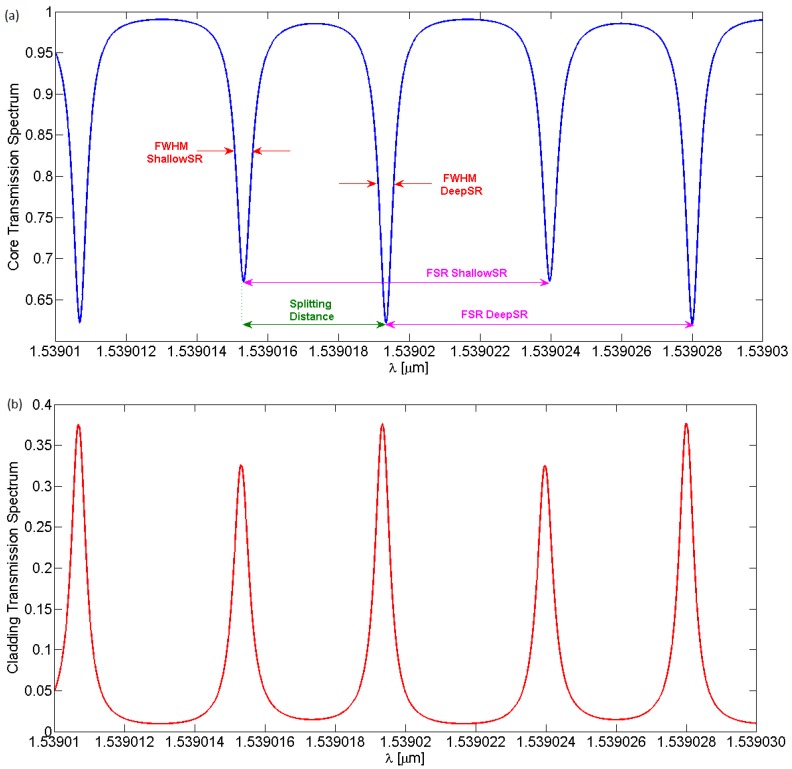
(**a**) LPGRR core spectrum with figures of merit labeled in; (**b**) LPGRR cladding spectrum.

Under these assumptions, the core and cladding spectral responses at the output of the LPGRR can be observed in Figure 5a,b.

**Figure 6:**
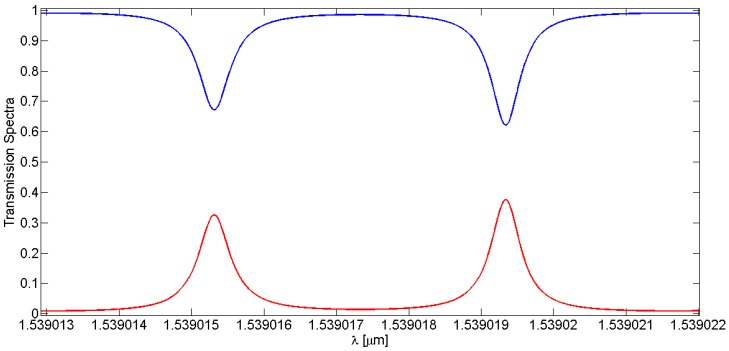
Zoom on the splitting resonances of core (blue curve) and cladding (red curve) spectral responses at ~1539 nm.

The peculiarity of this device is the presence of two resonance peaks, one deeper than the other in the core output spectrum and, dually, one higher than the other in the cladding one. This spectral feature is indicated as splitting resonances (SR), namely due to the combination of resonance effects of LPG and RR on the input signal. This effect can be clearly seen in Figure 6, where the LPGRR core and cladding transmission spectra are zoomed in around 1539 nm. The specific transmission spectra originate from the combination of the resonance characteristics of both RR and LPG. Note that the core and cladding modes outputs are dual because of their mutual power exchange.

RR filters can be described by specific figures of merit, such as the resonance width, defined as the full-width-at-half-maximum (FWHM) and the distance between resonance peaks, i.e., the free spectral range (FSR). A graphical estimation of these parameters has been performed for the LPGRR (see Figure 5a) and for a standard RR characterized by the same length. Numerical parameters are listed in Table 3. In particular, referring to the FWHM values, the width of the shallowest LPGRR core spectrum splitting resonance peak (Figure 5a) is 39.5% thinner than the standard RR resonances without any LPG. Furthermore, the width of the deepest LPGRR core spectrum splitting resonance peak is 44.2% thinner than standard RR resonances without LPG. Finally, the distance between the splitting resonances, i.e., labeled as splitting distance in Figure 5a,b, is equal to 4.03 pm.

### Parametric Analysis

**Figure 7:**
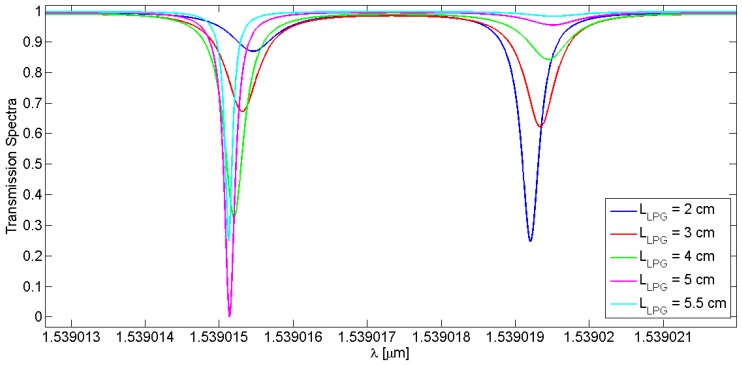
Transmission spectra of different LPGRRs characterized by the same core-to-cladding coupling coefficient and a LPG period of 311 µm, as a function of various grating lengths in the range from 2 cm to 5.5 cm.

**Figure 8:**
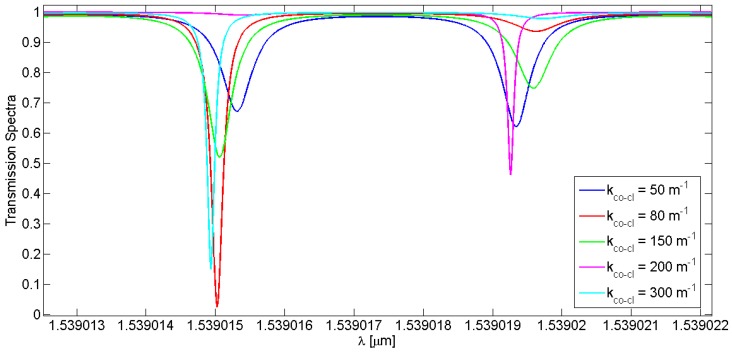
Transmission spectra of different LPGRRs characterized by the same length equal to 3 cm and period of 311 µm, as a function of the core-to-cladding coupling coefficients varied in the range of 50–300 m^−1^.

It is worth noting that the spectral responses, in particular the distance between the splitting peaks and their amplitudes, change by varying the LPG length and the core-to-cladding coupling coefficient, k_co-cl_. The simulations reported in Figure 7 and Figure 8 refer to the core spectrum centered at ~1539 nm, but still remain valid in the case of the cladding mode. Table 4 lists the calculated splitting resonance distance when the grating parameters are changed.

In Figure 7 and Table 4, the grating length of the LPG influences the distance between the splitting resonances, *d_spl_*; it increases linearly by increasing the grating length. Instead, a linear trend cannot be observed when the core and cladding mode splitting distance and coupling coefficient are considered, as also evidenced in Figure 8 and Table 4.

## 4. Overview of Sensing Applications

### Strain Sensitivity Prediction

**Figure 9:**
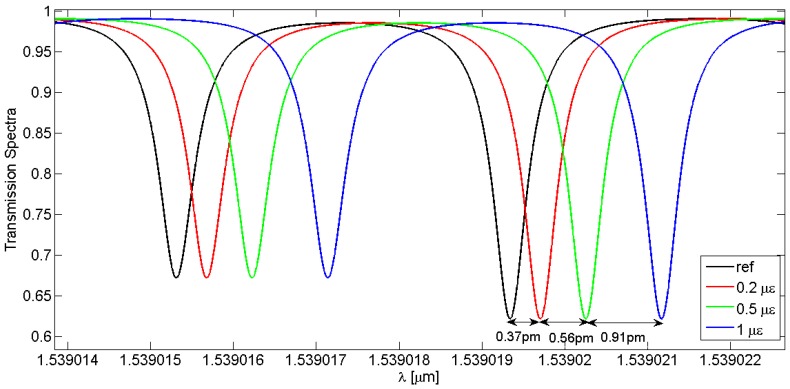
LPGRR spectral responses as a function of the applied strain in the range of 0–1 µɛ.

**Figure 10:**
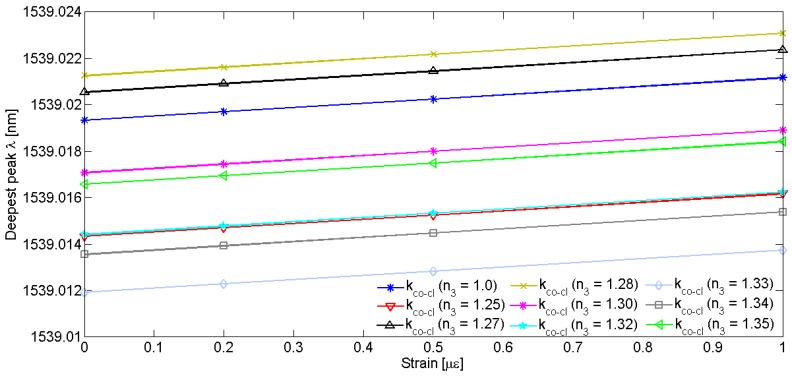
Wavelength shift of the deepest splitting resonance when various strains, ranging from 0 µɛ to 1 µɛ, are applied to an LPGRR with different core-to-cladding coupling coefficients.

In this paragraph the behaviour of the LPGRR device is investigated when a strain deformation is applied. The order of magnitude of the applied deformation is the microstrain (µɛ), where 1 µɛ is the strain producing a deformation of one part per million (10^−6^). The length along which the strain is applied is *L* as previously defined in Equation (6), so including the overall fiber length without the LPG and coupling region. In Figure 9, a red-shift of the resonance peaks occurs with increasing the applied strain. With a deformation ranging from 0 µɛ (i.e., the reference condition when the sensor is at rest) to 1 µɛ, the theoretical sensitivity has been estimated by tracking the wavelength shift of the deepest splitting resonance as a function of the applied strain. The influence of the external conditions on the strain sensing performances has also been evaluated by changing the core-to-cladding coupling coefficients as a function of the ambient RI n_3_ variations ranging from 1.25 to 1.35 RIU. In particular, it is worth specifying that the directional coupler parameters for the cladding mode, i.e., *κ_c_* and *τ_c_*, have been calculated as a function of the cover RI values as, differently from the core fundamental mode, the cladding mode is sensitive to localized RI perturbation in the cover medium. To this end, the cladding mode effective indexes calculated by FEM have been listed in Table 5 as a function of different values of n_3_. The sensitivity curves plotted in Figure 10 are theoretically represented by the parametric fitting equation *y* = *ax* + *b* whose values of slope and constant term are listed in Table 5 and depend from the values of the core-to-cladding coupling coefficients associated to the external operating conditions. It is worth noting that the coupling coefficients used for the sensitivity evaluation have an offset equal to 50 m^−1^ added to each of the theoretical values calculated as a function on the ambient RI, because it allows the best agreement between theoretical data and manufacturing specifications to be achieved.

As demonstrated in Figure 10 and Table 5, negligible slope variations of the sensitivity curves have resulted when the coupling coefficient is varied as a function of different cover RI. This means that the LPGRR-based strain sensor is practically immune to external environment conditions, when the temperature changes can be considered as stable and constant environmental parameters, and that the sensing performance, e.g., a strain sensitivity of ~1.8 × 10^−3^ nm/µɛ, can be still preserved when the sensor operates in different locations.

## 5. Conclusions

This paper explores, for the first time to the best of our knowledge, an optical device based on a long period fiber grating (LPG) inscribed in a section of a conventional fiberoptic racetrack resonator, namely an LPGRR. In particular, the LPGRR is fully and generically modelled by means of a TMM-based mathematical model which is a flexible and robust tool that allows optical and structural LPGRR changes to be easily simulated.

Spectral distributions of an open-loop LPG with a length of 3 cm and a period of 311 µm have been demonstrated, achieving a very good agreement between theory and experiments. Furthermore, the combination of the RR and the LPG generates specific transmittance spectra characterized by the presence of two resonance peaks, indicated as splitting resonances, separated by a spectral distance of 4.03 pm at ~1539 nm. This unique spectral operation has been simulated by varying LPG optical and geometrical features in order to perform a parametric analysis. In particular, the LPG length varied from 2 cm to 5.5 cm, resulting in an increase of the distance between the splitting resonances from 3.75 pm to 4.43 pm, respectively. Furthermore, by varying the core-to-cladding coupling coefficient from 50 m^−1^ to 300 m^−1^ the splitting resonances distance varies in a nonlinear way but in the proximity of 4 pm.

The LPGRR spectral response can be the subject of future studies and be employed for sensing applications. For example, the distance between the splitting resonances or the attenuation band shift can be used to perform sensing functionalities. In particular, the LPGRR-based strain sensing performances have been investigated in different environmental conditions with a core-to-cladding coupling coefficient varied as a function of the cover RI variations. A strain sensitivity of ~1.8 × 10^−3^ nm/µɛ can be theoretically achieved with an applied strain ranging from 0 μɛ to 1 μɛ. Finally, as negligible slope variations of the sensitivity curves have resulted, the sensing performances can be still preserved when the LPGRR operates in different locations. In conclusion, the LPGRR strain sensor can exhibit a linear operation and immunity from external environment conditions which can be extremely advantageous for several sensing applications.

## Figures and Tables

**Table 1:** SMF-28 and LPGRR parameters.

**SMF-28**			
Radius (µm)	RI		Effective RI
**Core**			
4.1	1.4489		1.446002
**Cladding**			
62.5	1.4440		1.441051
**Surrounding (*n*_3_)**			
150	1.0		-
**LPGRR**			
**Parameters**		**Values**	
*R*		3 cm	
*L_c_*		6.55 cm	
*L_s_*		6.6 cm	
*L_tot_*		32.05 cm	
*α*		4.6052 × 10^−4^ m^−1^	
*k_co-cl_*		50 m^−1^	
*DC_gap_*		10 µm	

**Table 2:** Coupling efficiency, transmission and coupling coefficients calculated with the Supermodes Theory in a 6.55 mm-long coupling region for both the core and cladding modes.

*τ* (m^−1^)	*κ* (m^−1^)	*η* (%)
Core		
0.71516	0.69896	48.85
Cladding		
0.96492	0.26255	6.89

**Table 3:** FWHM and FSR of the SRs of the LPGRR core spectrum and those of the resonance of a RR spectrum with the same length but without the LPG.

	*FWHM* (pm)	*FSR* (pm)
RR	0.86	8.66
Shallowest SR (LPGRR)	0.52	8.66
Deepest SR (LPGRR)	0.48	8.66

**Table 4:** Distance between the splitting resonances as a function of the LPG length and the core-to-cladding coupling coefficient.

*L_lpg_* (cm)	*d_spl_* (pm)	*k_co-cl_* (m^−1^)	*d_spl_* (pm)
2	3.75	50	4.03
3	4.04	80	4.62
4	4.27	150	4.54
5	4.37	200	4.61
5.5	4.43	300	4.74

**Table 5:** Optical and fitting parameters as a function of cover RI variations.

*n*_3_	*k_co-cl_* (m^−1^)	*a* (nm/µɛ)	*b*	*n^cl^_eff_*	*λ_res_* (nm)
1.00	0.0263	1.8253 × 10^−3^	1539.0193	1.441051	1539.8
1.25	0.0345	1.8290 × 10^−3^	1539.0143	1.441066	1535.1
1.27	0.0575	1.8220 × 10^−3^	1539.0205	1.441068	1534.5
1.28	0.0758	1.8403 × 10^−3^	1539.0212	1.441069	1534.2
1.30	0.1013	1.8266 × 10^−3^	1539.0171	1.441072	1533.2
1.32	0.0335	1.8266 × 10^−3^	1539.0144	1.441074	1532.6
1.33	0.0075	1.8273 × 10^−3^	1539.0119	1.441076	1531.9
1.34	0.0129	1.8280 × 10^−3^	1539.0136	1.441078	1531.4
1.35	0.03922	1.8280 × 10^−3^	1539.0166	1.44108	1530.7

## Abbreviations

LPG	Long Period Grating
RR	Ring Resonator
LPGRR	Long Period Grating Ring Resonator
RI	Refractive Index
TMM	Transfer Matrix Method
CMT	Coupled Mode Theory
PMC	Phase Matching Condition
SR	Splitting Resonances
